# A FRET-ICT Dual-Modulated Ratiometric Fluorescence Sensor for Monitoring and Bio-Imaging of Cellular Selenocysteine

**DOI:** 10.3390/molecules25214999

**Published:** 2020-10-28

**Authors:** Zongcheng Wang, Chenhong Hao, Xiaofang Luo, Qiyao Wu, Chengliang Zhang, Wubliker Dessie, Yuren Jiang

**Affiliations:** 1 College of Chemistry and Chemical Engineering, Central South University, Changsha 410083, China; wangzongch@huse.edu.cn (Z.W.); yaoyaowu@csu.edu.cn (Q.W.); zcl525@csu.edu.cn (C.Z.); 2 Hunan Engineering Technology Research Center for Comprehensive Development and Utilization of Biomass Resources, Hunan University of Science and Engineering, Yongzhou 425199, China; hao1248723618@163.com (C.H.); luoxf@huse.edu.cn (X.L.); wubliker@njtech.edu.cn (W.D.)

**Keywords:** FRET, ratiometric fluorescence sensor, cellular Sec, bio-imaging

## Abstract

Since the fluctuation of cellular selenocysteine (Sec) concentration plays an all-important role in the development of numerous human disorders, the real-time fluorescence detection of Sec in living systems has attracted plenty of interest during the past decade. In order to obtain a faster and more sensitive small organic molecule fluorescence sensor for the Sec detection, a new ratiometric fluorescence sensor **Q7** was designed based on the fluorescence resonance energy transfer (FRET) strategy with coumarin fluorophore as energy donor and 4-hydroxy naphthalimide fluorophore (with 2,4-dinitrobenzene sulfonate as fluorescence signal quencher and Sec-selective recognition site) as an energy acceptor. The sensor **Q7** exhibited only a blue fluorescence signal, and displayed two well distinguished emission bands (blue and green) in the presence of Sec with ∆λ of 68 nm. Moreover, concentrations ranging of quantitative detection of Sec of **Q7** was from 0 to 45 μM (limit of detection = 6.9 nM), with rapid ratiometric response, high sensitivity and selectivity capability. Impressively, the results of the living cell imaging test demonstrated **Q7** has the potentiality of being an ideal sensor for real-time Sec detection in biosystems.

## 1. Introduction

Selenium (Se) as a vital micronutrient element plays an allimportant role in physiological and pathological processes of humans, such as maintaining redox balance, activating immunoreaction, preventing cancer development, and protecting inflammation [1,2,3,4]. It has been confirmed that different chemical forms of Se exist in human systems, including selenocysteine (CysSeH, Sec), hydrogen selenide (H_2_Se), selenoproteins (SePs), selenoglutathione (GSeH), cysteine selenopersulfide (CysSSeH), charged Sec-tRNA, etc. [5,6,7]. Among them, Sec, an analogue of cysteine (Cys) with the thiol group replaced by selenium-containing selenol group, seems to serve as the main functional form of numerous Se-containing species in living systems, and is known as the specifical building block for the incorporation of selenoproteins (SePs) in the active site of enzymes [8,9]. SePs, such as thioredoxin reductases (TrxRs) and glutathione peroxidases (GPxs), show a variety of biological functions related to a series of human disorders, such as inflammation, cardiovascular, cancers, cognitive decline, Keshan and Kashin–Beck disease [10,11,12]. Hence, to obtain more precise physiological and pathological information of Sec, it is very urgent and important to develop methods with efficiency and reliability for the identification of Sec in biosystems [13].

To date, there are numerous detection methods for the determination of Sec, such as thin-layer chromatography, high performance liquid chromatography, capillary electrophoresis inductively coupled plasma mass spectrometry, and gas chromatography [14,15]. Nevertheless, these methods need complicated pretreatment or destruction of biological samples. In comparison, fluorescence imaging methods exhibit latent capability to serve as more widely utilized tools for the detection of biological relative analytes, since they can offer the higher selectivity and sensitivity, faster response, nondestructiveness, as well as high resolution [16,17]. However, the design of Sec-specific fluorescent sensors is still challenging because of the existing more abundant chemically similar biothiols, including glutathione (GSH), homocysteine (Hcy) and cysteine (Cys), which may bring latent interference due to similar chemical properties [18,19]. Hence, the research of fluorescent sensors for the selective identification of Sec progressed slowly [20]. Impressively, after several years of effort, the first generation of Sec-selective fluorescence sensors with high physiological applicability has been developed based on the difference of pKa between biothiols (~8.3) and Sec (~5.8) [21,22]. Thereafter, the development of Sec-selective fluorescent sensor advanced rapidly [23]. Even so, many reported sensors seem to be limited in the practical application of detection of Sec in vivo due to the poor sensitivity as shown in Table S1 (limit of detection = 9~62 nM) [21,22,24,25]. It is well known that ratiometric fluorescent sensors always display high sensitivity, especially low detection limit, since the built-in correction of the dual-emission band of ratiometric response can circumvent the interference brought by the efficiency of the instrument, and the concentration of the sensor [26]. Despite that several ratiometric Sec-selective fluorescence sensors have been reported previously [27,28,29], in order to explore clearer pathophysiological roles of Sec in biological systems, it is urgent to develop the novel fluorescent sensors with high selectivity and sensitivity (especially with low detection limit), as well as rapid detection response.

Herein, we described a new ratiometric fluorescence sensor **Q7**, based on the fluorescence resonance energy transfer (FRET) strategy for the highly selective and sensitive identification of cellular Sec. The sensor **Q7** has the capability of selectively detecting the concentration fluctuation of Sec within 4 min and displays a dual emission ratiometric response without the interference brought by other bio-relative thiols (including Cys). Additionally, taking advantage of the built-in correction of two well-distinguished emission bands, the **Q7** sensor could determinate the concentration of Sec within 4 min in a wide linear range (0–45 μM) with a lower detection limit (6.9 nM). Moreover, the living cell fluorescence imaging test results indicated that the sensor **Q7** could effectively and efficiently identify the concentration fluctuation of cellular Sec.

## 2. Results and Discussion

### 2.1. Design of Ratiometric Sensor

**Scheme 1:**
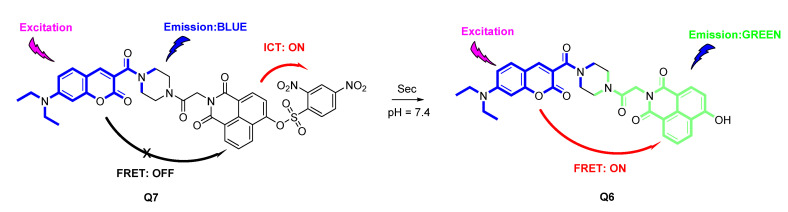
The proposed response mechanism of ratiometric fluorescent sensor **Q7** toward Sec.

**Scheme 2:**
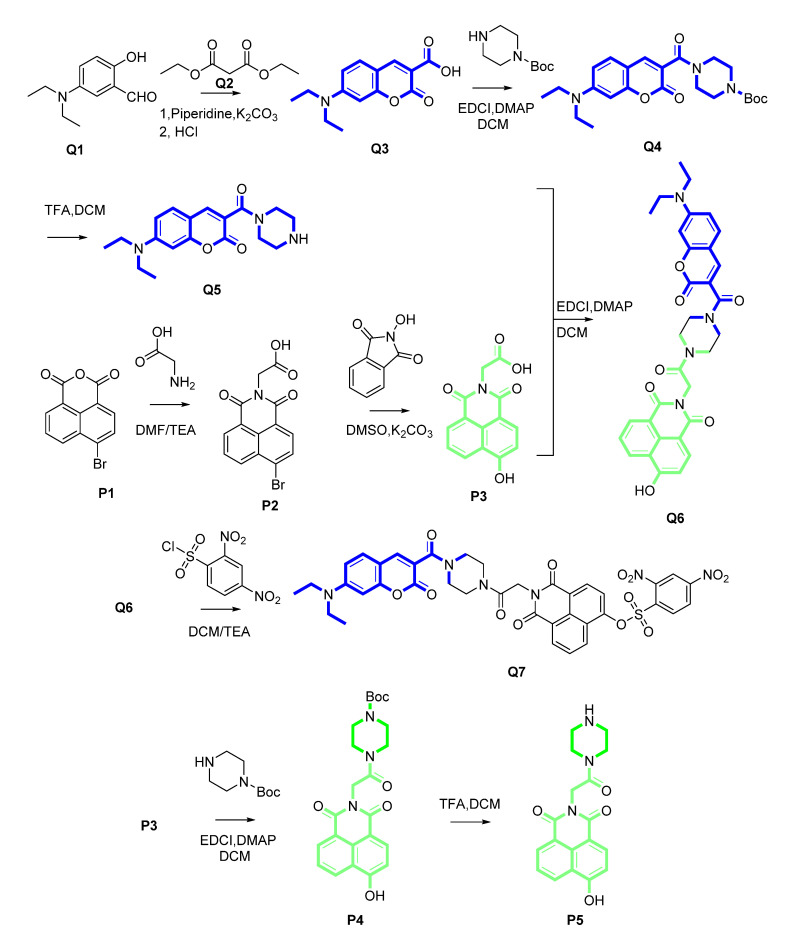
The synthetic route of energy donor **Q5**, energy acceptor **P5**, fluorophore **Q6** and the ratiometric fluorescent sensor **Q7**.

In the design of a ratiometric fluorescent sensor, as shown in Scheme 1, we employed coumarin as a donor fluorophore and hydroxy naphthalimide fluorophore as an energy receptor to construct ratiometric dye **Q6** through a piperazine linker. As for the recognition moiety, a 2,4-dinitrobenzene sulfonate ester moiety was chosen to serve as a Sec-specific response site over biothiols for the difference of nucleophilicity of Sec and biothiols in living systems. Additionally, since the strong electron withdrawing effect of the response site, the 2,4-dinitrobenzenesulfonyl moiety would theoretically modulate the internal charge transfer (ICT) effect of **Q6** dye resulting in the signal change of the naphthalimide receptor. Herein, we speculated that the sensor **Q7** itself showed only a blue emission signal (ascribed to the coumarin moiety) due to ICT quenching effect of 2,4-dinitrobenzene sulfonate ester group. However, the addition of Sec with **Q7** would cleave the sulfonate ester group with green fluorescence signal recovery (ascribed to naphthalimide fluorophore) via ICT effect blocked and FRET effect restored, as shown in Scheme 2, accordingly achieving ratiometric fluorescence identification of Sec theoretically under physiological conditions. **Q6** dye was prepared by amidation of piperazine with carboxyl group functionalized coumarin and 4-hydroxy naphthalimide. Sensor **Q7** was synthesized with **Q6**, 2,4-dinitrobenzenesulfonyl chloride, and triethylamine as the deacid reagent. The energy donor **Q5**, energy acceptor **P5**, fluorophore **Q6** and the ratiometric fluorescent sensor **Q7** were characterized by ^1^H NMR, ^13^C NMR, and HRMS (refer to Figures S1–S12 of the Supporting Information).

### 2.2. Spectral Properties of Q7

To evaluate the detection properties of the ratiometric fluorescence sensor **Q7** toward Sec under simulated physiological conditions, we first examined the absorption and fluorescence spectra of **Q6** dye (1 μM) and sensor **Q7** (1 μM) in PBS buffer (10 mM, pH 7.4, with 1% DMSO).

**Figure 1:**
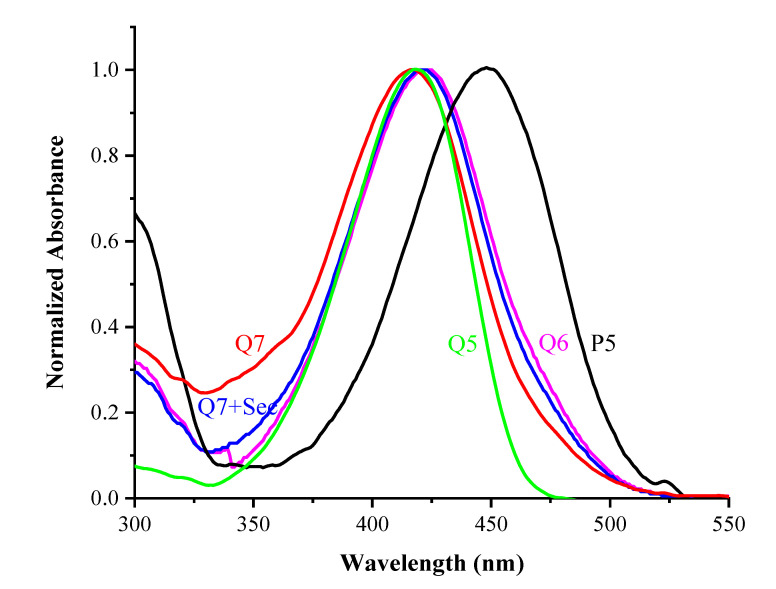
Normalized absorption curve of 1 μM **P5** (black), **Q5** (green), **Q6** (pink), **Q7** (red), and **Q7** reacting with 10 μM Sec (blue) in DMSO/PBS = 1:99 (*v*/*v*).

As shown in Figure 1, the **Q6** dye displayed the maximum absorption at 422 nm (pink), while the maximum absorbance of energy acceptor **P5** peaked in the region of longer wavelength around 448 nm (black). Meanwhile, the maximum absorption peak of **Q7** sensor was around 418 nm (red), which was similar to that of donor fluorophore **Q5** (green). Additionally, the solution of sensor treated with Sec exhibited an absorption peak around 421 nm, which was similar to that of **Q6**. All the results of UV-vis spectrum analysis indicated the hydroxy group of **Q6** had been blocked by 2,4-dinitrobenzene sulfonate ester and might be recovered by the reaction with Sec.

**Figure 2:**
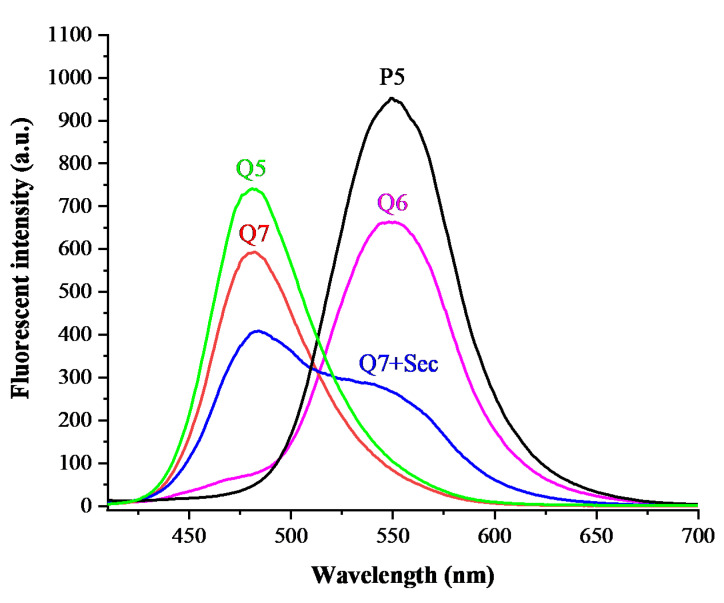
Fluorescence spectra of 1 μM **P5** (black), **Q5** (green), **Q6** (pink), **Q7** (red), and **Q7** reacting with 10 μM Sec (blue) in DMSO/PBS = 1:99 (*v*/*v*), excited at 400 nm.

At the same time, we tested the fluorescence emission spectrum of solutions of **Q7**, **Q7** after addition of Sec, **Q6**, **Q5** and **P5** under the same conditions. As depicted in Figure 2, the solution of **Q7** displayed only a blue fluorescence signal and the maximum emission was to be similar to that of **Q5**, while the **Q6** dye showed a green emission signal with the maximum emission similar to that of **P5** (excited at 400 nm). After treated **Q7** with Sec (10 μM), the blue signal at 482 nm decreased, the green signal at 550 nm increased (blue) and ∆λ was 68 nm. The spectrogram demonstrated that the 2,4-dinitrobenzene sulfonate ester of **Q7** sensor could be cleaved by the addition of Sec with a ratiometric fluorescence response. Next, as shown in Figure S13 of the Supporting Information, HPLC analysis was employed to prove the recovery of **Q6** dye after the addition of Sec into **Q7** solution. The HPLC spectra of **Q7** solution after treatment of Sec (10 μM) displayed a new signal (4.033 min, blue) with the retention time similar to that of **Q6** (4.042 min, black) as shown in Figure S13, which clarified the determination mechanism of the sensor **Q7** toward Sec.

### 2.3. The Sensitivity Studies

**Figure 3:**
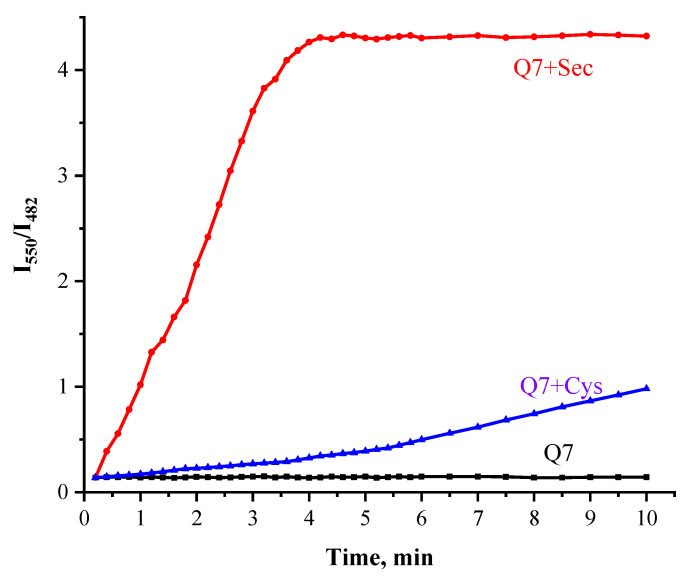
Effect of time on Fluorescent intensity ratios I_550_/I_482_ of 1 µM **Q7** (black), **Q7** reacting with 100 μM Sec (red) and **Q7** reacting with 1 mM Cys (blue) in DMSO/PBS = 1:99 (*v*/*v*), excited at 400 nm.

As for the sensitivity studies of Sec detection by Q7 sensor, we firstly examined the optimal reaction time. Given the instability of Sec, (Sec)_2_ and DTT were mixed in equal molar amounts in PBS buffer to obtain fresh analyte. The green emission of **Q7** after addition with Sec (100 μM) increased significantly, while the blue signal decreased rapidly in the initial stage. Both of them reached stability within about 4 min (Figure 3) indicating Q7 owned a rapider ratiometric fluorescence response property when compared with the previously reported same type of Sec fluorescence sensors [22,30]. However, the green emission of **Q7** after being added with Cys (1 mM) did not increase significantly in 4 min.

**Figure 4:**
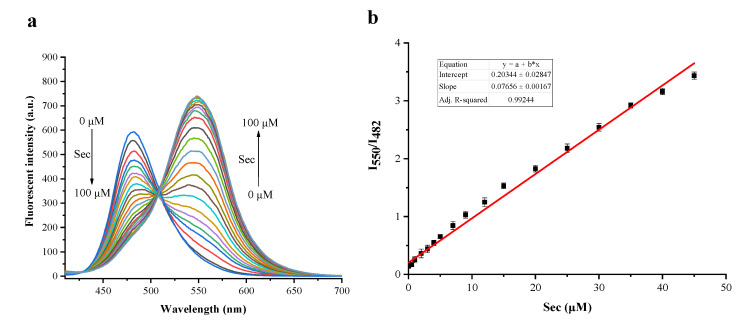
(**a**) Fluorescence intensity variation of 1 µM **Q7** to different Sec concentrations (0, 0.5, 1, 2, 3, 4, 5, 7, 9, 12, 15, 20, 25, 30, 35, 40, 45, 50, 60, 70, 80, 90, 100 µM). (**b**) The linear relationship of the fluorescent intensity ratios I_550_/I_482_ of 1 µM **Q7** and Sec concentrations, excited at 400 nm.

We further determined the detection sensitivity of **Q7** to Sec. Figure 4a shows that the emission intensity of **Q7** increased at 550 nm and declined gradually at 482 nm after the treatment of various concentrations of Sec (0–100 μM) for 4 min. Interestingly, a reasonable linear relationship was obtained between the fluorescence ratio (I_550_/I_482_) and Sec concentrations (0–45 μM), and the equation of linear regression was determined as I_550_/I_482_ = 0.07656 [Sec] μM + 0.20344 with R^2^ = 0.99244. Herein, the detection limit of sensor **Q7** for Sec was determined as low as about 6.9 nM (LOD = 3σ/k), which showed that the sensor **Q7** owned a wide linear range and higher sensitivity for quantitative detection of Sec compared with the previously reported same type of Sec fluorescence sensors [24,25].

### 2.4. The Selectivity and Anti-Interference Properties

**Figure 5:**
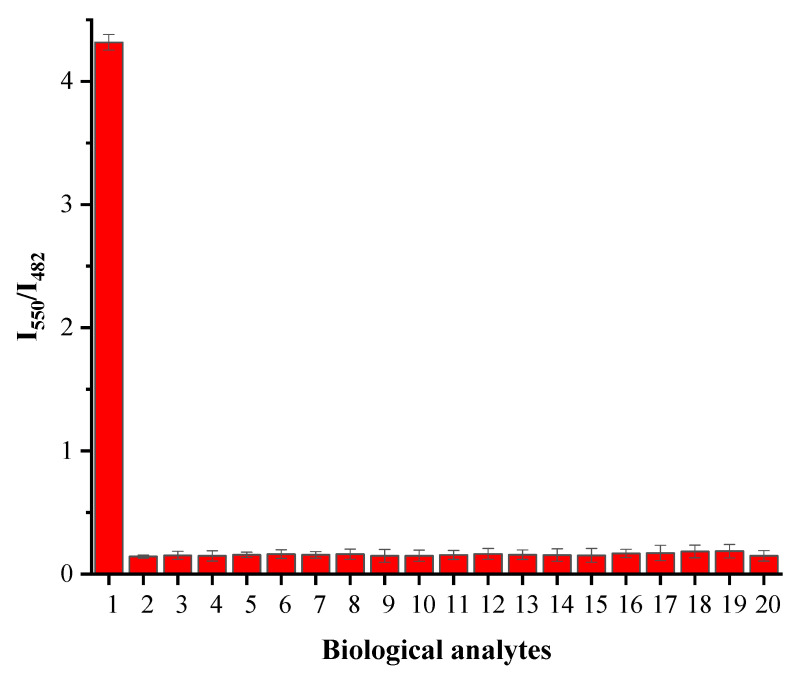
Fluorescent intensity ratios I_550_/I_482_ responses of 1 μM **Q7** to biological analytes (1 mM). Legend: (1) Sec; (2) Blank; (3) Hcy; (4) GSH; (5) Cys; (6) Pro; (7) Glu; (8) Asp; (9) Phe; (10) Thr; (11) Val, (12) Leu, (13) Arg; (14) Ile; (15) Ser; (16) Trp; (17) Lys; (18) His; (19) Na_2_SeO_3_; (20) Na_2_Se. Excitation at 400 nm. Each data point was detected after mixing for 4 min.

The selectivity is an important index to evaluate the sensing properties of a new fluorescence sensor, and thereby we further investigated the selectivity of detecting Sec by **Q7** sensor under simulated conditions. As shown in Figure 5, the addition of other biological active substances (including Pro, Glu, Asp, Phe, Thr, Val, Leu, Arg, Ile, Ser, Trp, Lys, His, Hcy, Cys, GSH, Na_2_SeO_3_ and Na_2_Se) could not render a significant change of emission intensity of sensor solution, which demonstrated that this newly synthesized **Q7** sensor owned a good sensitivity toward Sec over biothiols, representative amino acids, and other selenocompounds.

Additionally, in order to evaluate the capability of practical application under simulated conditions of **Q7**, we further examined the anti-interference ability of **Q7** by the method of recording the intensity change of sensor solution which was treated with other biological analytes in the presence of Sec. As shown in Figure S14, no remarkable intensity changes had been observed, indicating **Q7** sensor had a latent capability of being applied in a complex detection environment.

### 2.5. Cell Experiments

Inspired by the excellent sensing properties and anti-interference ability, we next investigated the cytotoxicity and fluorescence imaging capability of Sec determination by **Q7** sensor. As shown in Figure S15, the cell viability was maintained at over 80% when pre-cultured by 0–10 μM sensor solutions, which meant that the sensor **Q7** exhibited barely obvious cytotoxicity and was able to be applied in a living cell model. Although the concentration of Sec is very low in normal human cells, its concentration will increase in the presence of some physiological diseases, such as thyroid cancer, which can be detected and recognized by fluorescence sensors [17,28]. To investigate fluorescence detection capability of **Q7** in living cells, a rationmetric fluorescence imaging test was carried out.

**Figure 6:**
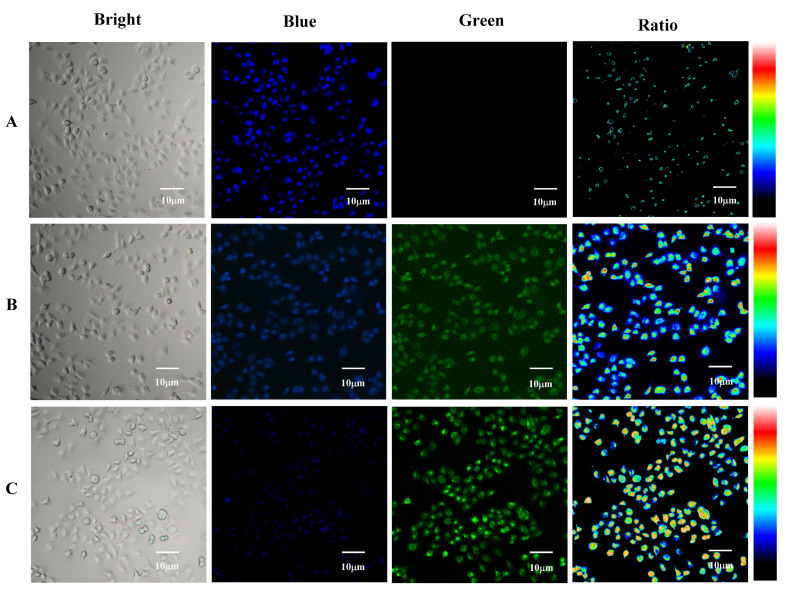
Fluorescence images of living A549 cells was measured with a bright, blue and green channel. Blue channel, excitation from 361 to 389 nm and emission from 430 to 490 nm; green channel, excitation from 465 to 495 nm and emission from 512 to 558; ratio, a rationmetric imaging generated from green and blue channels. (**A**) A549 cells were incubated with 1 μM **Q7** for 6 h, (**B**) A549 cells were incubated with 10 μM (Sec)_2_ for 6 h, then with 1 μM **Q7** for 10 min, (**C**) A549 cells were incubated with 10 μM (Sec)_2_ for 6 h, then with 1 μM **Q7** for 30 min. Scale bar: 10 μm.

As shown in Figure 6, the control group of cells pretreated with 1 μM of **Q7** itself for 30 min, displayed only a blue emission signal (Panel A), which suggested that **Q7** could not respond to the cellular biothiols. However, the test group of cells pre-cultured with 10 μM of (Sec)_2_ (reacted with biothiols to generate Sec in cells) for 6 h and successive addition of **Q7** for 10 min showed an obvious green emission signal with the blue signal declined (Panel B). Additionally, the group was pretreated with 10 μM of (Sec)_2_ and subsequently cultured by **Q7** for 30 min, displaying stronger green emission accompanying weak blue fluorescence intensity and strong rationmetric imaging intensity (Panel C). All the results of the fluorescence imaging test indicated the sensor **Q7** had the potential of being applied to ratiometric fluorescence detection of Sec in living cells.

## 3. Materials and Methods

### 3.1. Materials and Equipment

Procurement of all commercial chemicals was from commercial suppliers without further purification. A Bruker Advance III HD400 spectrometer (Zurich, Switzerland) was used to record ^1^H NMR (400 MHz) and ^13^C NMR (100 MHz) spectra (with TMS as internal standard). An Agilent 6540 mass spectrometer (Palo Alto, CA, USA) was applied to measure high resolution mass spectrum of intermediates and final products. SHIMADZU UV-2600 and SHIMADZU RF-6000 fluorescence spectrometers (Kyoto, Japan) were used for obtaining absorbance and fluorescence spectrum.

### 3.2. Synthesis of the Fluorescent Sensor

The energy donor **Q5**, energy acceptor **P5**, fluorophore **Q6** and the ratiometric fluorescent sensor **Q7** were synthesized as shown in Scheme 2.

The compound **Q3** was prepared based on the method reported previously [31]. The compounds 4-(Diethylamino) salicylaldehyde (**Q1**, 1.93 g, 10 mmol), diethyl malonate (**Q2**, 1.60 g, 10 mmol) and anhydrous K_2_CO_3_ (2.07 g, 15 mmol) were mixed and stirred to reflux in the ethanol solution (50 mL) with piperidine (425 mg, 5 mmol) as a catalyst. After the reaction was completed as shown by thin layer chromatography (TLC), the reaction mixture was cooled to room temperature and poured into 100 mL of water following the addition of 1M HCl solution to acidify the solution with yellowish red precipitate formed. The precipitate was filtrated and washed to neutral with water. After drying, the orange product (1.77 g, yield: 68%) was not further purified and used directly in the next step.

The aforementioned compound **Q3** (1.31 g, 5 mmol), 1-boc-piperazine (1.12 g, 6 mmol), 1-ethyl-3(3-dimethylpropylamine) carbodiimide (EDCI, 4.78 g, 25 mmol) and 4-dimethylaminopyridine (DMAP, 305 mg, 2.5 mmol) were added in a flask with anhydrous dichloromethane (40 mL) and stirred at room temperature for 2 h. After the solvent was removed, the residue was subsequently purified by silica gel chromatography column with dichloromethane (DCM)/ Methanol (MeOH) (20:1, *v*/*v*) as eluent [29]. The compound **Q4** was prepared as a yellowish crystal (1.35 g, yield: 63%).

The compound **Q4** (858 mg, 2 mmol) was added into the 15 mL of mixed solution of DCM/TFA (2:1, *v*/*v*) and then further stirred for 2 h at room temperature. After the raw material had been consumed, the solvent was removed and the residue was then purified by silica gel chromatography column with DCM/MeOH (20:1, *v*/*v*) as eluent [32]. The compound **Q5** was prepared as a yellow crystal (539 mg, yield: 82%). ^1^H NMR (400 MHz, DMSO-*d*_6_) δ 9.03 (s, 1H), 8.03 (s, 1H), 7.52 (d, *J* = 9 Hz, 1H), 6.76 (dd, *J* = 9, 2 Hz, 1H), 6.56 (d, *J* = 2 Hz, 1H), 3.77–3.57 (m, 4H), 3.46 (q, *J* = 7 Hz, 4H), 3.20–3.05 (m, 4H), 1.13 (t, *J* = 7 Hz, 6H). ^13^C NMR (100 MHz, DMSO-*d*_6_) 164.81, 159.00, 157.25, 151.95, 145.35, 130.79, 115.21, 109.99, 107.61, 96.73, 44.68, 12.76. ESI-MS (C_18_H_24_N_3_O_3_)^+^: 330.18063, calcd for (C_18_H_24_N_3_O_3_)^+^: 330.18177.

According to the methodology reported previously, the compound **P2** was synthesized [33]. Specifically, the compound **P1** (2.76 g, 10 mmol), glycine (900 mg, 12 mmol) and triethylamine (1.20 g, 12 mmol) was added into 50 mL N,N-dimethylformamide. The reaction mixture was heated to 105 °C and stirred for 5 h until the reaction completed. Subsequently, the mixture was cooled to room temperature and poured into 150 mL cold H_2_O with brown precipitate formed (2.19 g, yield: 66%). After drying, the brown product was not purified and used directly in the next step.

The compound **P2** (1.66 g, 5 mmol), N-hydroxyphthalimide (3.26 g, 10 mmol), and anhydrous potassium carbonate (6.15 g, 15 mmol) were added into DMSO (50 mL) and stirred at 100 °C for 2 h. The reaction liquid was poured into massive water to form precipitation. The precipitate was filtrated and subsequently purified by a silica gel chromatography column with DCM/MeOH (20:1, *v*/*v*) as eluent [34]. The compound **P3** was synthesized as a yellowish crystal (976 mg, yield: 72%).

The compound **P3** (271 mg, 1 mmol), compound **Q5** (329 mg, 1 mmol), EDCI (955 mg, 5 mmol) and DMAP, (61 mg, 0.5 mmol) were added into anhydrous DCM (20 mL) and stirred at room temperature for 2 h. The solvent was removed and the residue was subsequently purified by silica gel chromatography column with DCM/MeOH (20:1, *v*/*v*) as eluent. The compound **Q6** was prepared as yellowish crystal (337 mg, yield: 58%). ^1^H NMR (400 MHz, DMSO-*d*_6_) δ 11.94 (s, 1H), 8.53 (d, *J* = 8 Hz, 1H), 8.44 (d, *J* = 7 Hz, 1H), 8.33 (d, *J* = 8 Hz, 1H), 8.02 (d, *J* = 8 Hz, 1H), 7.75 (t, *J* = 8 Hz, 1H), 7.45 (d, *J* = 8 Hz, 1H), 7.16 (d, *J* = 8 Hz, 1H), 6.68 (s, 1H), 6.51 (s, 1H), 4.91 (d, *J* = 7 Hz, 2H), 3.74–3.53 (m, 12H), 1.09 (t, *J* = 6 Hz, 6H). ^13^C NMR (100 MHz, DMSO-*d*_6_) 165.77, 164.88, 163.93, 163.20, 160.97, 159.00, 157.11, 151.72, 144.54, 134.19, 131.73, 130.60, 129.72, 126.08, 122.88, 121.94, 116.09, 112.74, 110.49, 109.81, 107.61, 96.71, 44.63, 41.43, 12.72. ESI-MS (C_32_H_31_N_4_O_7_)^+^: 583.21928, calcd for (C_32_H_30_N_4_O_7_)^+^: 583.21927.

The compound **Q6** (291 mg, 0.5 mmol) and 120 mg of TEA were dissolved in 10 mL of precooled anhydrous DCM (0 °C). A total of 5 mL of anhydrous DCM with 2, 4-dinitrobenzene sulfonyl chloride (160 mg, 0.6 mmol) was subsequently added into the reaction mixture dropwise, and further stirred for 1 h at room temperature until the reaction was accomplished. The solvent was removed and the remaining residue was then finally purified by silica gel chromatography column with DCM/MeOH (20:1, *v*/*v*) as eluent. A yellow solid product **Q7** was obtained (304 mg, yield: 75%). ^1^H NMR (400 MHz, DMSO-*d*_6_) δ 9.16 (d, *J* = 2 Hz, 1H), 8.63–8.57 (m, 2H), 8.50 (d, *J* = 8 Hz, 1H), 8.43 (dd, *J* = 8, 1 Hz, 1H), 8.37 (d, *J* = 8 Hz, 1H), 8.09–7.91 (m, 2H), 7.71 (d, *J* = 8 Hz, 1H), 7.51 (d, *J* = 8 Hz, 1H), 6.75 (d, *J* = 8 Hz, 1H), 6.56 (s, 1H), 4.98 (d, *J* = 8 Hz, 2H), 3.73 (q, *J* = 7 Hz, 4H), 3.60–3.39 (m, 8H), 1.13 (t, *J* = 7 Hz, 6H). ^13^C NMR (100 MHz, DMSO-*d*_6_) δ 165.30, 163.24, 162.64, 158.97, 157.14, 152.23, 151.79, 149.16, 148.59, 134.38, 132.52, 132.04, 130.91, 130.65, 129.37, 129.30, 128.26, 125.35, 122.59, 122.02, 121.84, 120.77, 116.10, 109.90, 107.61, 96.78, 44.65, 41.84, 12.77. ESI-MS (C_38_H_33_N_6_O_13_S)^+^: 813.18201, calcd for (C_38_H_33_N_6_O_13_S)^+^: 813.18263.

The compound **P4** was prepared as a yellowish crystal with the obtained compound **P3** (271 mg, 1 mmol), 1-boc-piperazine (223 mg, 1.2 mmol), EDCI (955 mg, 5 mmol) and DMAP (61 mg, 0.5 mmol) as the raw material based on the **Q4** method [32,35]. Thereafter, the compound **P5** was prepared by the reaction of **P4** (220 mg, 0.5 mmol) with 4 mL of DCM/TFA (1:1, *v*/*v*) for 2 h (room temperature), and then the compound **P5** (122 mg, yield: 72%) was obtained by purification with silica gel chromatography column (DCM/MeOH (20:1, *v*/*v*) as eluent). ^1^H NMR (400 MHz, DMSO-*d*_6_) δ 12.48 (s, 1H), 9.49 (s, 1H), 8.55 (dd, *J* = 8, 1 Hz, 1H), 8.44 (dd, *J* = 7, 1 Hz, 1H), 8.33 (d, *J* = 8 Hz, 1H), 7.79–7.71 (m, 1H), 7.23 (d, *J* = 8 Hz, 1H), 4.94 (s, 2H), 3.91 (s, 2H), 3.69 (s, 2H), 3.26 (s, 2H), 3.13 (s, 2H). ^13^C NMR (100 MHz, DMSO-*d*_6_) 166.01, 163.98, 163.23, 161.43, 134.28, 131.74, 129.75, 126.02, 122.98, 121.88, 119.04, 112.42, 110.58, 43.26, 42.99, 41.79, 41.22, 38.85. ESI-MS (C_18_H_18_N_3_O_4_)^+^: 340.12924, calcd for (C_18_H_18_N_3_O_4_)^+^: 340.12973.

### 3.3. Spectrum Analysis

The required amount of solid product **Q7** was dissolved in DMSO to prepare the stock solution of sensor **Q7** with the final concentration of 100 μM. All the absorbance and fluorescence emission spectra were recorded by a UV-vis spectrophotometer and fluorescence spectrometer. The spectrum analysis of sensor, sensor solution with addition of Sec, energy donor, and energy acceptor was carried out by 1.0 cm quartz cell. The test samples were prepared as following: The sensor stock solution was diluted to 1 μM (containing 1% DMSO) by PBS (10 mM, pH 7.40), and various amounts of freshly prepared Sec solutions (equimolar (Sec)_2_ reacted with dithiothreitol (DTT) in PBS at 37 °C) were added into colorimetric volumetric flasks with further 4 min reaction at 25 °C before spectrum analysis [36].

### 3.4. Anti-Interference Assays

The stock solutions of test biological active analytes with potential interference (including Pro, Glu, Asp, Phe, Thr, Val, Leu, Arg, Ile, Ser, Trp, Lys, His, Hcy, Cys, GSH, Na_2_SeO_3_ and Na_2_Se) were prepared by dissolving the exact mass of the above compounds into double distilled water with final concentrations of 1 mM. The selectivity tests were carried out by mixing the sensor solution with different species, respectively, for 4 min before measurement. The competition assays were conducted by reaction of the sensor solution with different species in the presence of Sec after 4 min.

### 3.5. Cell Imaging

A549 cells (human lung cancer cells), were cultured in dishes in 1640 medium with 10% FBS, 1% penicillin, and 1% streptomycin at 37 °C in a CO_2_ cell incubator with 5% CO_2_ [37]. After being incubated for 24 h, the cell experiments were carried out. Cytotoxicity of sensor **Q7** was determined by standard MTT assay. (Sec)_2_ was added as the source of Sec. After 1 μM sensor was added, the cells were incubated for a different time and washed with PBS (pH = 7.4). Cell images were carried out by a Nikon Ni-U fluorescence microscope.

## 4. Conclusions

In this study, a new FRET-ICT dual-modulated ratiometric fluorescence sensor **Q7** was designed and prepared by introducing 2,4-dinitrobenzene sulfonate ester to a FRET ratiometric fluorophore **Q6**, which was constructed by coumarin as a donor fluorophore with hydroxy naphthalimide fluorophore as energy receptor through a piperazine linker. The sensor **Q7** exhibited only a blue fluorescence signal, and displayed two well-distinguished emission bands (blue and green) in the presence of Sec with ∆λ of 68 nm. Moreover, **Q7** has the capability of quantitative detection of Sec at concentrations ranging from 0 to 45 μM (limit of detection = 6.9 nM), whose detection limit is lower than those of the reported sensors [19,21,22,24,25,35,36,37]. The response time of **Q7** to Sec is 4 min, which is faster than those of the reported sensors [19,22,25,35,37]. In addition, the sensor **Q7** can be used for rationmetric fluorescence imaging in living cells. Herein, we speculated that this novel FRET-ICT dual-modulated ratiometric fluorescence sensor will be able to be applied to a wider field of biomedical diagnostics.

## Supplementary Materials

The following are available online, Table S1. The performance parameters of some reported Sec fluorescent sensors and detection method, Figure S1: ^1^H NMR spectrum of compound **Q5** (DMSO-*d_6_*), Figure S2 ^13^C NMR spectrum of compound **Q5** (DMSO-*d_6_*), Figure S3 HRMS spectrum of compound **Q5**, Figure S4 ^1^H NMR spectrum of compound **Q6** (DMSO-*d_6_*), Figure S5 ^13^C NMR spectrum of compound **Q6** (DMSO-*d_6_*), Figure S6 HRMS spectrum of compound **Q6**, Figure S7 ^1^H NMR spectrum of compound **Q7** (DMSO-*d_6_*), Figure S8 ^13^C NMR spectrum of compound **Q7** (DMSO-*d_6_*), Figure S9 HRMS spectrum of compound **Q7**, Figure S10 ^1^H NMR spectrum of compound **P5** (DMSO-*d_6_*), Figure S11 ^13^C NMR spectrum of compound **P5** (DMSO-*d_6_*), Figure S12 HRMS spectrum of compound **P5**, Figure S13 HPLC spectrum of compound **Q6** (black), **Q7** (pink) and treating **Q7** with Sec (blue). Mobile phase: MeOH: H_2_O = 7:3, UV detection wavelength: 420nm, Figure S14 Fluorescent intensity ratios I_550_/I_482_ responses of 1 μM **Q7** at 614 nm to Sec (40 μM) in the presence of various biological analytes (1 mM) in PBS (10 mM, pH 7.40, containing 1% DMSO as cosolvent). Legend: (1) Blank; (2) Hcy; (3) GSH; (4) Cys; (5) Pro; (6) Glu; (7) Asp; (8) Phe; (9) Thr; (10) Val, (11) Leu, (12) Arg; (13) Ile; (14) Ser; (15) Trp; (16) Lys; (17) His; (18) Na_2_SeO_3_; (19) Na_2_Se. Excitation at 400 nm. Each data was obtained 4 min after mixing, Figure S15 Cytotoxicity of A549 cells by standard MTT assay in the presence of sensor **Q7** (0~10 μM) at 37 °C.

## References

[1] Zadeh M.H., Farsani G.M., Zamaninour N. (2019). Selenium status after Roux-en-Y gastric bypass: Interventions and recommendations. Obes. Surg..

[2] Frączek A., Pasternak K. (2013). Selenium in medicine and treatment. J. Elementol..

[3] Wu D., Chen L.Y., Kwon N., Yoon J. (2016). Fluorescent probes containing selenium as a guest or host. Chem.

[4] Brinkman M., Buntinx F., Muls E., Zeegers M.P. (2006). Use of selenium in chemoprevention of bladder cancer. Lancet Oncol..

[5] Kuganesan M., Samra K., Evans E., Singer M., Dyson A. (2019). Selenium and hydrogen selenide: Essential micronutrient and the fourth gasotransmitter?. Intens. Care Med. Exp..

[6] Shang N.N., Wang X.F., Shu Q.M., Wang H., Zhao L.N. (2019). The functions of selenium and selenoproteins relating to the liver diseases. J. Nanosci. Nanotechno..

[7] Reddy P.S., Metanis N. (2016). Small molecule diselenide additives for in vitro oxidative protein folding. Chem. Commun..

[8] Peeler J.C., Falco J.A., Kelemen R.E., Abo M., Chartier B.V., Edinger L.C., Chen J.J., Chatterjee A., Weerapana E. (2020). Generation of recombinant mammalian selenoproteins through genetic code expansion with photocaged selenocysteine. ACS Chem. Biol..

[9] Pessione E., Pessione A., Mangiapane E. (2014). Selenium and selenoproteins: An overview on different biological systems. Curr. Protein Pept. Sci..

[10] Zhang X., Zhang L., Zhu J.H., Cheng W.H. (2016). Nuclear selenoproteins and genome maintenance. IUBMB Life.

[11] Pillai R., Uyehara-Lock J.H., Bellinger F.P. (2014). Selenium and selenoprotein function in brain disorders. IUBMB Life.

[12] Loscalzo J. (2014). Keshan disease, selenium deficiency, and the selenoproteome. New Engl. J. Med..

[13] Zhang P.P., Ding Y., Liu W.M., Niu G.L., Zhang H.Y., Ge J.C., Wu J.S., Li Y.Q., Wang P.F. (2018). Red emissive fluorescent probe for the rapid detection of selenocysteine. Sens. Actuators B.

[14] Bednarik A., Kuta J., Vu D.L., Ranglova K., Hrouzek P., Kanicky V., Preisler J. (2018). Thin-layer chromatography combined with diode laser thermal vaporization inductively coupled plasma mass spectrometry for the determination of selenomethionine and selenocysteine in algae and yeast. J. Chromatogr. A.

[15] Thosaikham W., Jitmanee K., Sittipout R., Maneetong S., Chantiratikul A., Chantiratikul P. (2014). Evaluation of selenium species in selenium-enriched pakchoi *(Brassica chinensis Jusl var parachinensis (Bailey) Tsen & Lee)* using mixed ion-pair reversed phase HPLC-ICP-MS. Food Chem..

[16] Zhang L., Kai X.N., Zhang Y.R., Zheng Y.G., Xue Y.S., Yin X.X., Zhao J. (2018). A reaction-based near-infrared fluorescent probe that can visualize endogenous selenocysteine in vivo in tumor-bearing mice. Analyst.

[17] Kong F.P., Hu B., Gao Y., Xu K.H., Pan X.H., Huang F., Zheng Q.L., Chen H., Tang B. (2015). Fluorescence imaging of selenol in HepG2 cell apoptosis induced by Na_2_SeO_3_. Chem. Commun..

[18] Guo Y.D., Luo Y., Wang N., Tang M.G., Xiao J.C., Chen S.W., Wang J.Y. (2020). Au nanoparticle-based probe for selenol in living cells and selenium-rich tea and rice. Talanta.

[19] Feng W.Y., Li M.X., Sun Y., Feng G.Q. (2017). Near-infrared fluorescent turn-on probe with a remarkable large stokes shift for imaging selenocysteine in living cells and animals. Anal. Chem..

[20] Maeda H., Katayama K., Matsuno H., Uno T. (2006). 3′-(2,4-Dinitirobenzenesulfonyl)-2′,7′-dimethyl-fluorescein as a fluorescent probe for selenols. Angew. Chem. Int. Edit..

[21] Zhang S.R., Wang Q., Liu X.W., Zhang J.J., Yang X.F., Li Z., Li H. (2018). Sensitive and selective fluorescent probe for selenol in living cells designed via a pK(a) shift strategy. Anal. Chem..

[22] Dai C.G., Wang J.L., Song Q.H. (2016). Red fluorescent probes based on a BODIPY analogue for selective and sensitive detection of selenols in solutions and in living systems. J. Mater. Chem. B.

[23] Liu Y.N., Feng X.H., Yu Y.N., Zhao Q.Y., Tang C.H., Zhang J.M. (2020). A review of bioselenol-specific fluorescent probes: Synthesis, properties, and imaging applications. Anal. Chim. Acta.

[24] Zhang B.X., Ge C.P., Yao J., Liu Y.P., Xie C.H., Fang J.G. (2015). Selective selenol fluorescent probes: Design, synthesis, structural determinants, and biological applications. J. Am. Chem. Soc..

[25] Li M.X., Feng W.Y., Zhai Q.S., Feng G.Q. (2017). Selenocysteine detection and bioimaging in living cells by a colorimetric and near-infrared fluorescent turn-on probe with a large stokes shift. Biosens. Bioelectron..

[26] Zhao X.J., Wang C., Yuan G.Q., Ding H.Y., Zhou L.Y., Liu X.G., Lin Q.L. (2019). A dual-site modulated FRET-based two-photon ratiometric fluorescent probe for tracking lysosomal pH changes in living cells, tissues and zebrafish. Sens. Actuators B.

[27] Tian Y., Xin F.Y., Gao C.C., Jing J., Zhang X.L. (2017). Ratiometric fluorescent imaging for endogenous selenocysteine in cancer cell matrix. J. Mater. Chem. B.

[28] Luo X.Z., Wang R., Lv C.Z., Chen G., You J.M., Yu F.B. (2020). Detection of selenocysteine with a ratiometric near-infrared fluorescent probe in cells and in mice thyroid diseases model. Anal. Chem..

[29] Zhao X.J., Yuan G.Q., Ding H.Y., Zhou L.Y., Lin Q.L. (2020). A TP-FRET-based fluorescent sensor for ratiometric visualization of selenocysteine derivatives in living cells, tissues and zebrafish. J. Hazard. Mater..

[30] Zhang L., Shi Y.F., Sheng Z.J., Zhang Y.R., Kai X.N., Li M.Y., Yin X.X. (2019). Bioluminescence imaging of selenocysteine in vivo with a highly sensitive probe. ACS Sens..

[31] Yuan L., Lin W.Y., Xie Y.N., Chen B., Song J.Z. (2012). Fluorescent detection of hypochlorous acid from turn-on to FRET-based ratiometry by a HOCl-mediated cyclization reaction. Chem. Eur. J..

[32] Shu W., Yan L.G., Liu J., Wang Z.K., Zhang S., Tang C.C., Liu C.Y., Zhu B.C., Du B. (2015). Highly selective fluorescent probe for the sensitive detection of inorganic and organic mercury species assisted by H_2_O_2_. Ind. Eng. Chem. Res..

[33] Liang S.C., Yu H., Xiang J., Yang W., Chen X.H., Liu Y.B., Gao C., Yan G.P. (2012). New naphthalimide modified polyethylenimine nanoparticles as fluorescent probe for DNA detection. Spectrochim. Acta Part A.

[34] Chen Y.C., Zhu C.C., Cen J.J., Li J., He W.J., Jiao Y., Guo Z.J. (2013). A reversible ratiometric sensor for intracellular Cu^2+^ imaging: Metal coordination-altered FRET in a dual fluorophore hybrid. Chem. Commun..

[35] Zhang H.Y., Li M.X., Feng W.Y., Feng G.Q. (2018). Rapid and selective detection of selenocysteine with a known readily available colorimetric and fluorescent turn-on probe. Dyes Pigments.

[36] Wang Z.C., Zheng H.H., Zhang C.L., Tang D.F., Wu Q.Y., Dessie W., Jiang Y.R. (2020). A red emissive fluorescent turn-on sensor for the rapid detection of selenocysteine and its application in living cells imaging. Sensors.

[37] Sun Q., Yang S.H., Wu L., Dong Q.J., Yang W.C., Yang G.F. (2016). Detection of intracellular selenol-containing molecules using a fluorescent probe with near-zero background signal. Anal. Chem..

