# Supplementary material for: A FRET-ICT Dual-Modulated Ratiometric Fluorescence Sensor for Monitoring and Bio-Imaging of Cellular Selenocysteine
Source: Molecules. 2020 Oct 28;25(21):4999. doi: 10.3390/molecules25214999 (PMC7663636; doi:10.3390/molecules25214999)
Supplement: Supplementary file 1 [file molecules-25-04999-s001.pdf]

A FRET-ICT dual-modulated Ratiometric Fluorescence Probe for Monitoring and Bio-imaging of Cellular Selenocysteine

Supplementary Materials

Zongcheng Wang[1,2], Chenhong Hao[2], Xiaofang Luo[2], Qiyao Wu[1], Chengliang Zhang[1], Wubliker Dessie[2], Yuren Jiang[1,*]

[1] College of Chemistry and Chemical Engineering, Central South University, Changsha, 410083, China

[2] Hunan Engineering Technology Research Center for Comprehensive Development and Utilization of Biomass Resources, Hunan University of Science and Engineering, Yongzhou, 425199, China

Correspondence to:

*Yuren Jiang

College of Chemistry and Chemical Engineering, Central South University, Changsha 410083, Hunan, China

Telephone number: 86-0731-86781389

Fax number: 86-0731-88859988

E-mail: jiangyr@mail.csu.edu.cn

Table S1. The performance parameters of some reported Sec fluorescent sensors and detection method

Sensors or method	Structure	λ_{Ex} /nm	λ_{Em} /nm	λ_{Stokes} /nm	LOD /nM	Response time/min	Bioimaging	Ref
This work	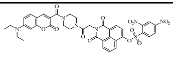	422	550/482	128/60	6.9	4	A549	-
TLC	-	-	-	-	38	-	-	[14]
HPLC-ICP-MS	-	-	-	-	60	-	-	[15]
WY-Sec	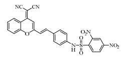	478	670	192	60	10	A549/ Mice	[19]
MC-Sec	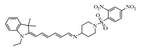	550	593	43	68	3	SMMC-7721	[21]
Sel-p1	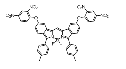	594	663	69	16	15	MCF-7/Mice	[22]
Sel-p2	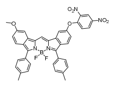	594	655	61	9	15	MCF-7/Mice	[22]
Sel-green	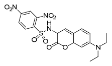	370	502	132	62	3	HepG2	[24]
GQ-Sec	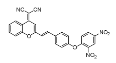	560	706	146	62	5	HeLa	[25]
BTHC- Sec	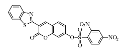	453	493	40	47	30	HeLa	[35]
YZ-A4	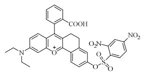	550	614	64	11.2	3	A549	[36]
GF-Sec	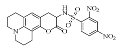	380	535	155	18	8	A549/ zebrafish	[37]

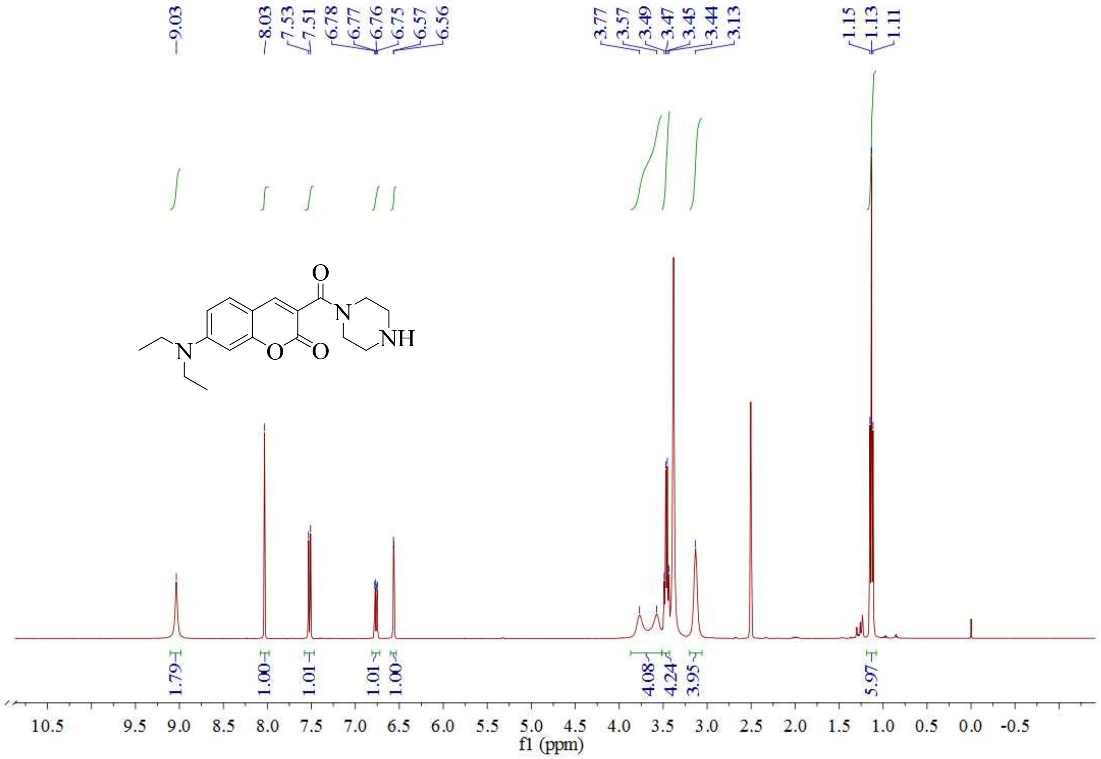

Figure S1 ^{1}H NMR spectrum of compound **Q5** (DMSO-*d*$_6$)

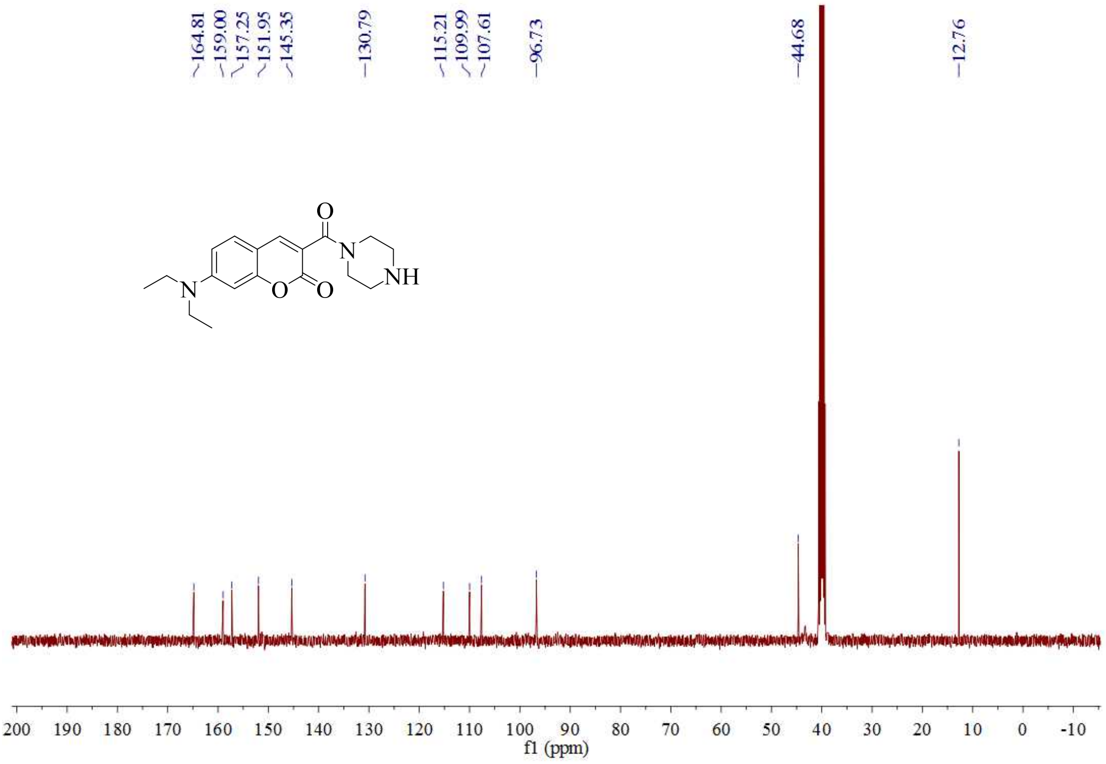

Figure S2 ^{13}C NMR spectrum of compound **Q5**(DMSO-*d*$_6$)

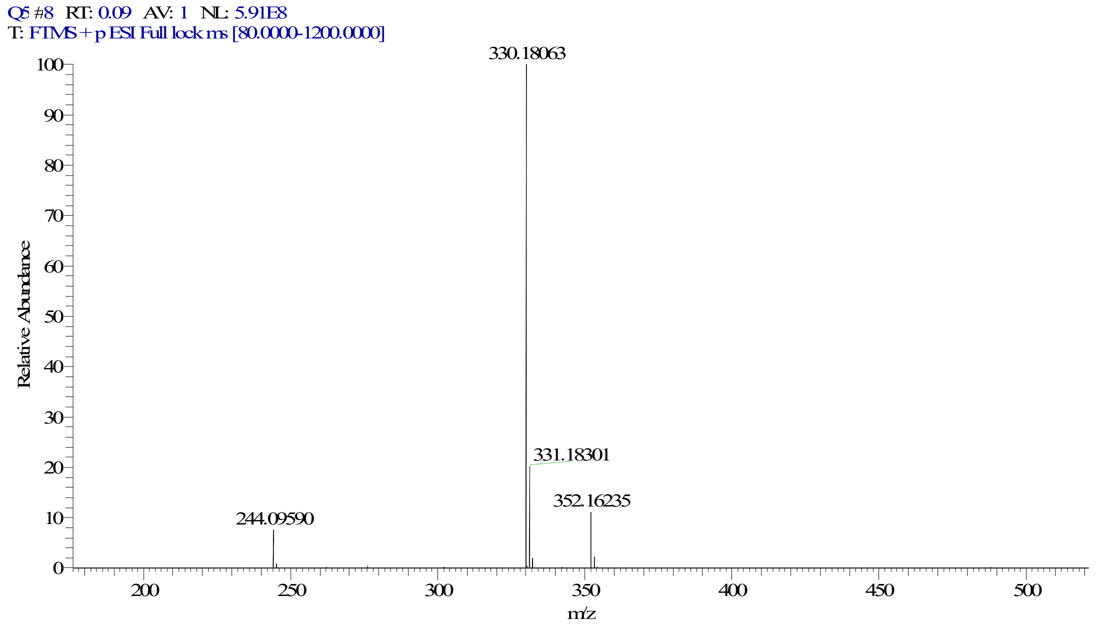

Figure S3 HRMS spectrum of compound **Q5**

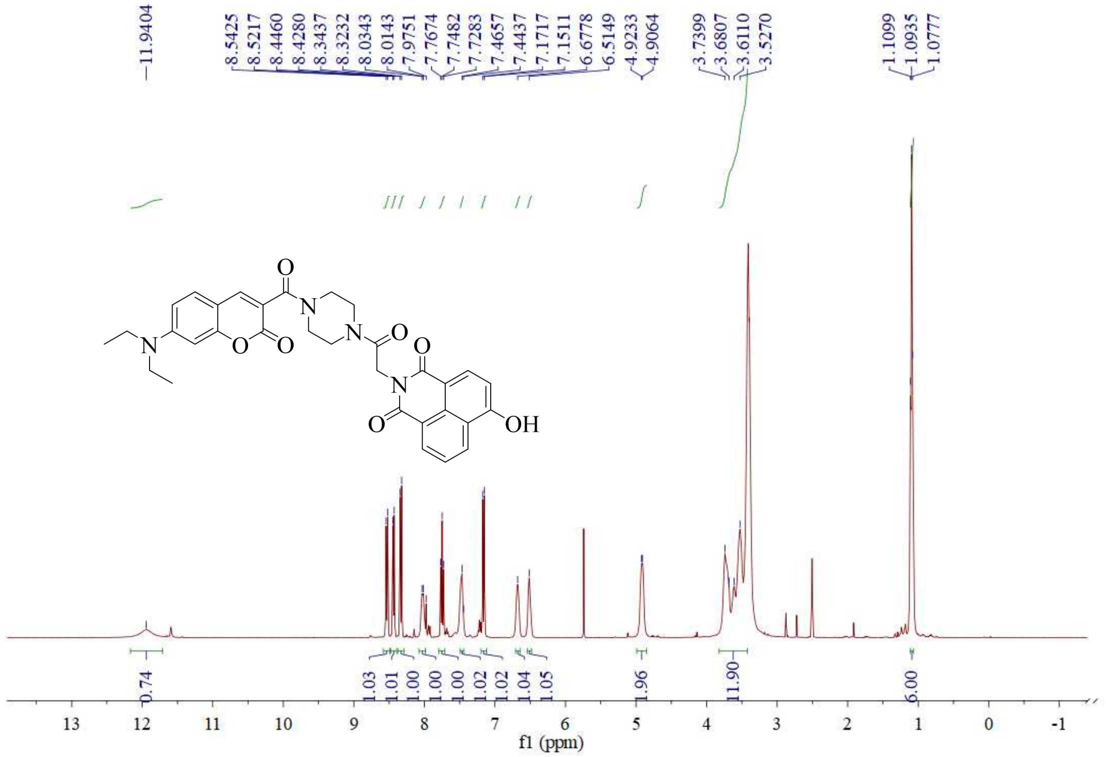

Figure S4 ^{1}H NMR spectrum of compound **Q6** (DMSO-d_6)

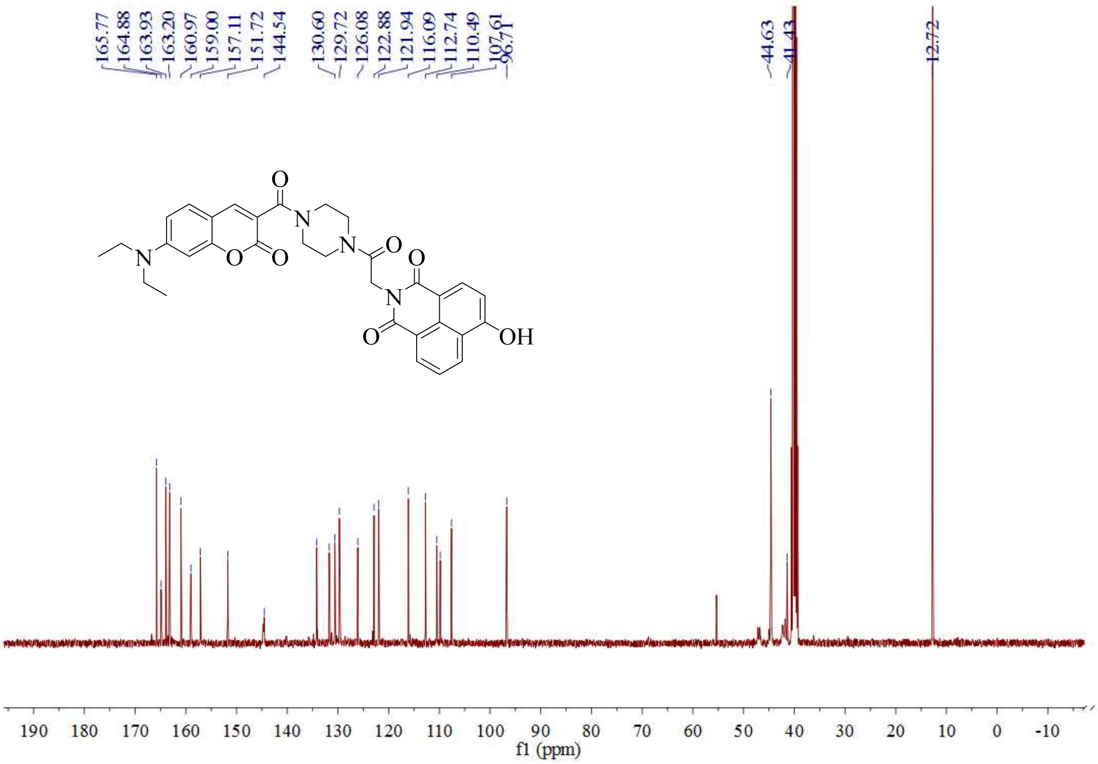

Figure S5 ^{13}C NMR spectrum of compound **Q6** (DMSO-*d*$_6$)

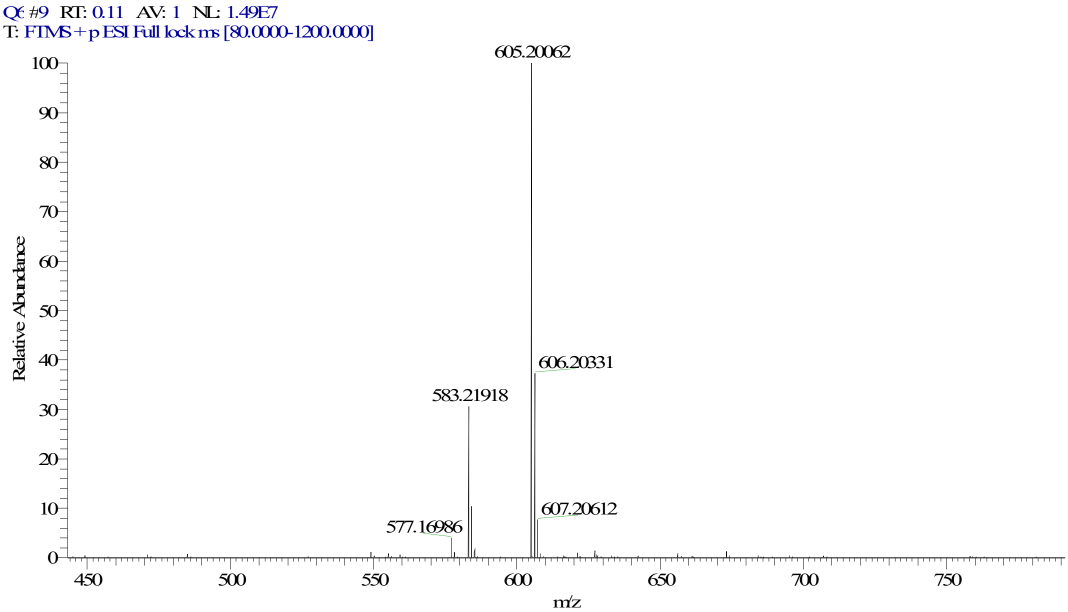

Figure S6 HRMS spectrum of compound **Q6**

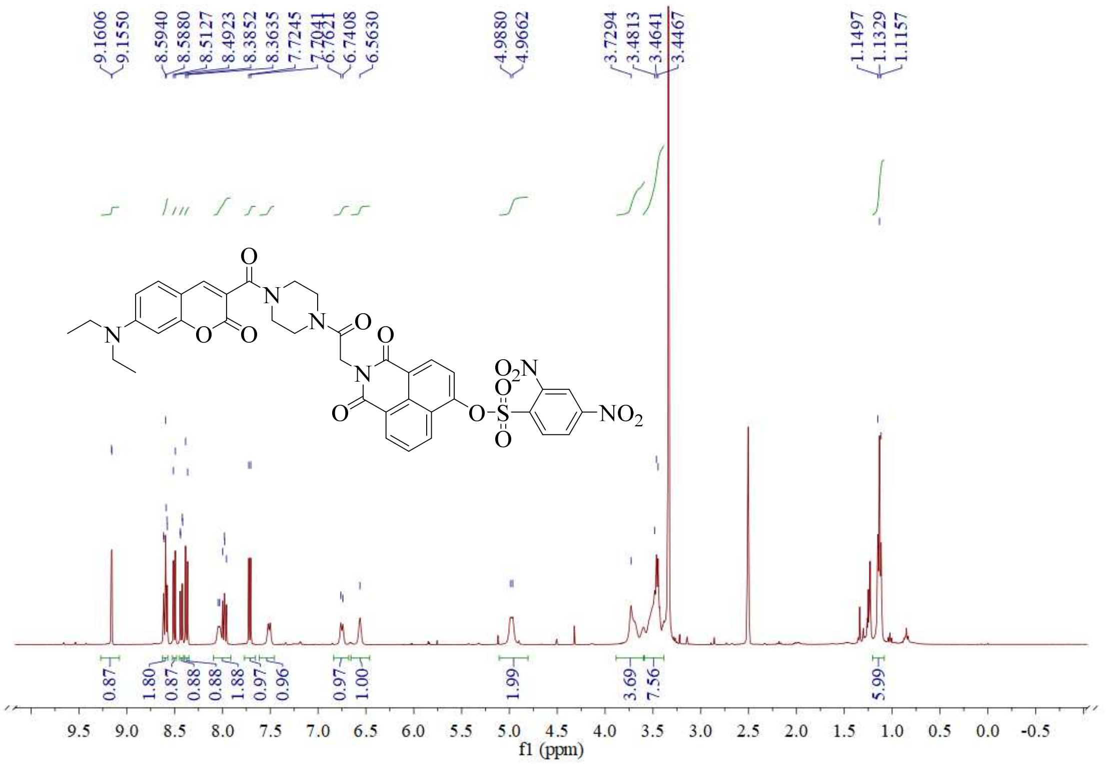

Figure S7 ^{1}H NMR spectrum of compound **Q7** (DMSO-*d6*)

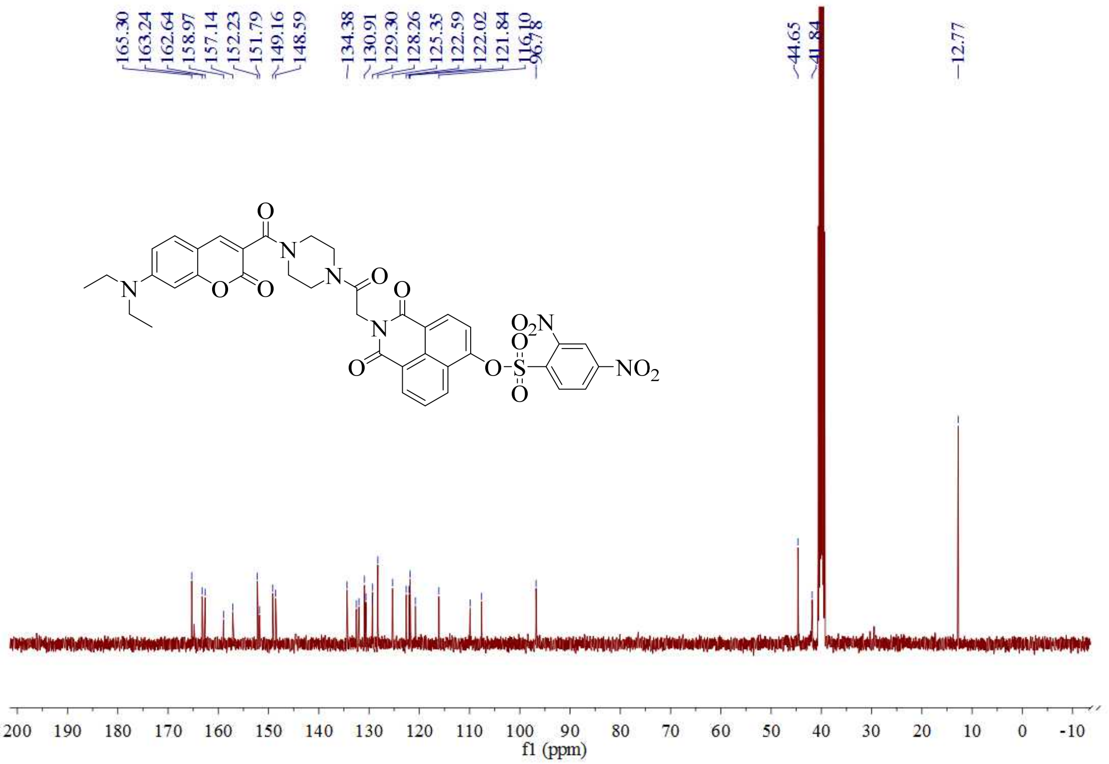

Figure S8 ^{13}C NMR spectrum of compound **Q7** (DMSO-*d6*)

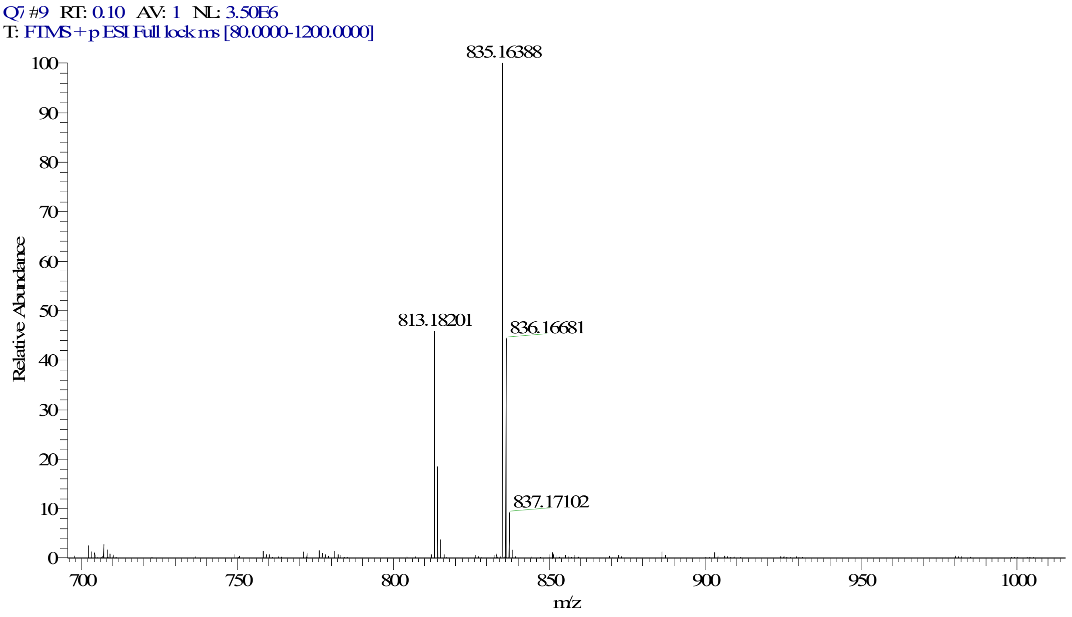

Figure S9 HRMS spectrum of compound **Q7**

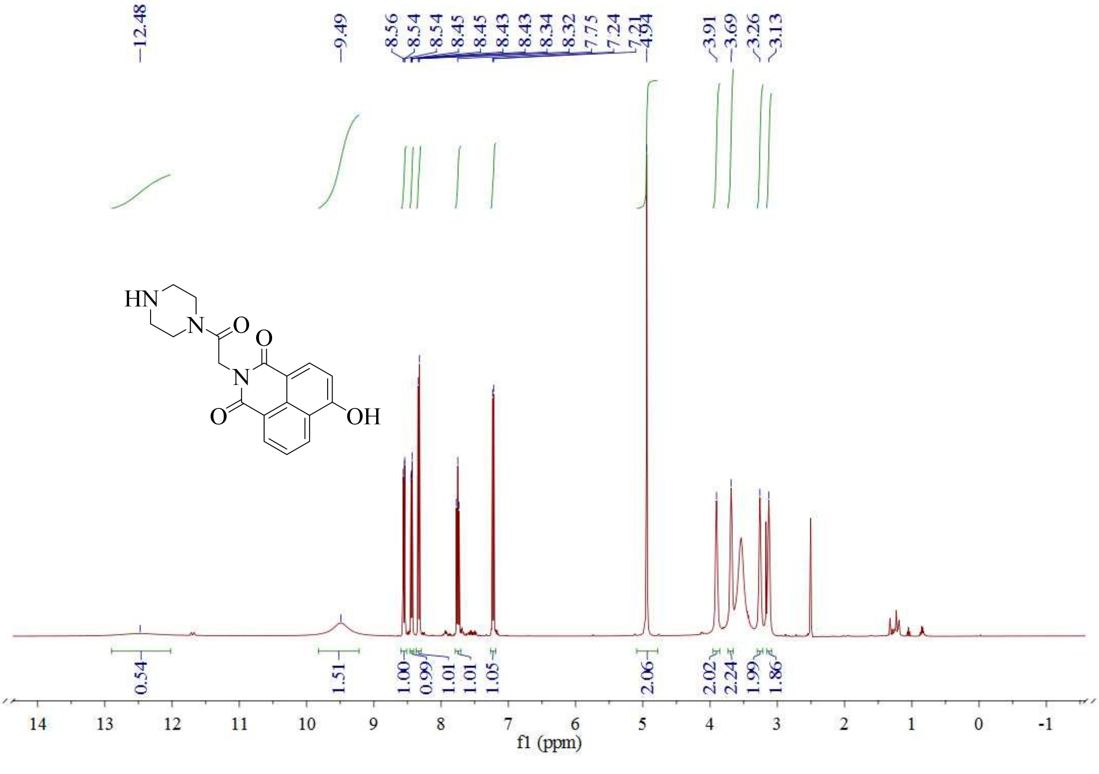

Figure S10 ^{1}H NMR spectrum of compound **P5**(DMSO-d_6)

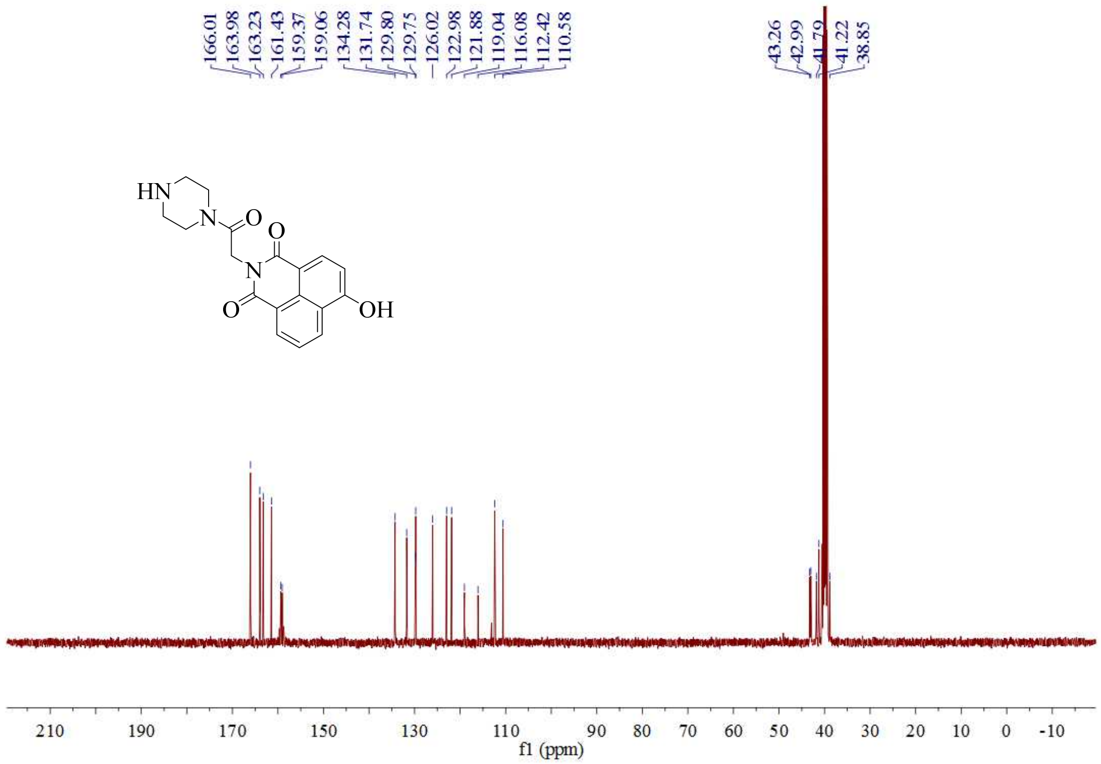

Figure S11 ^{13}C NMR spectrum of compound **P5** (DMSO-*d*$_6$)

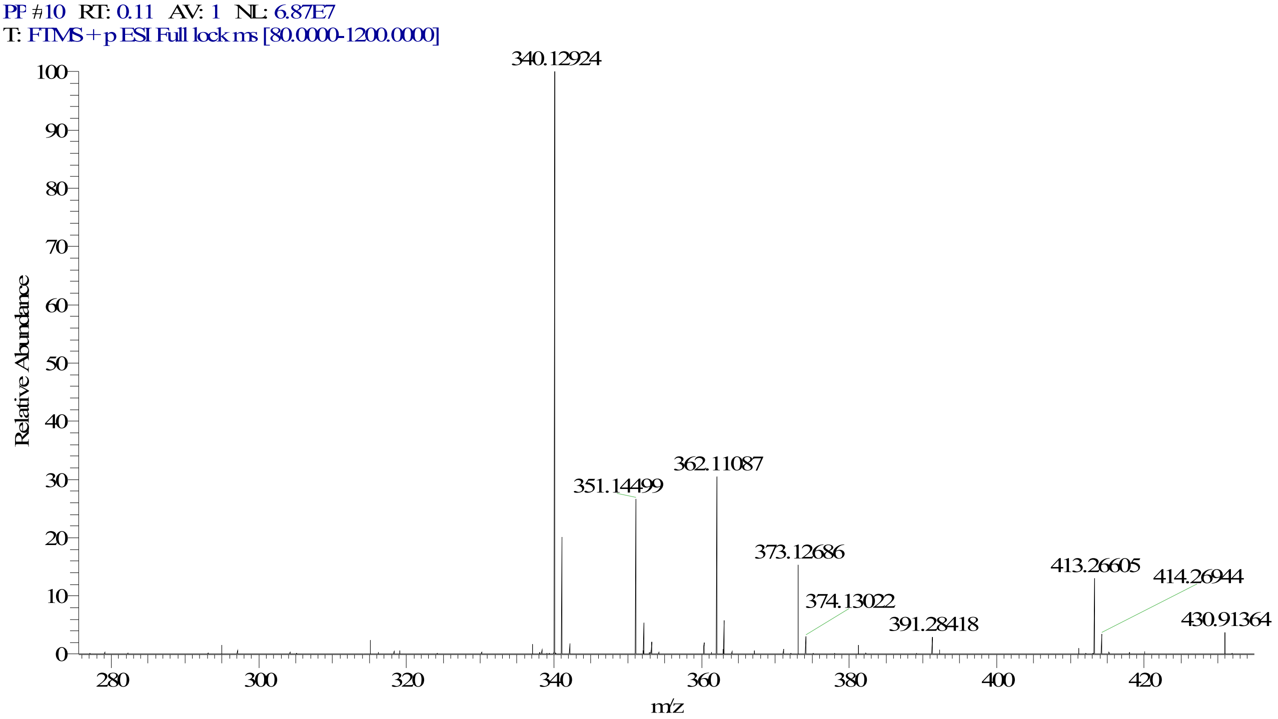

Figure S12 HRMS spectrum of compound **P5**

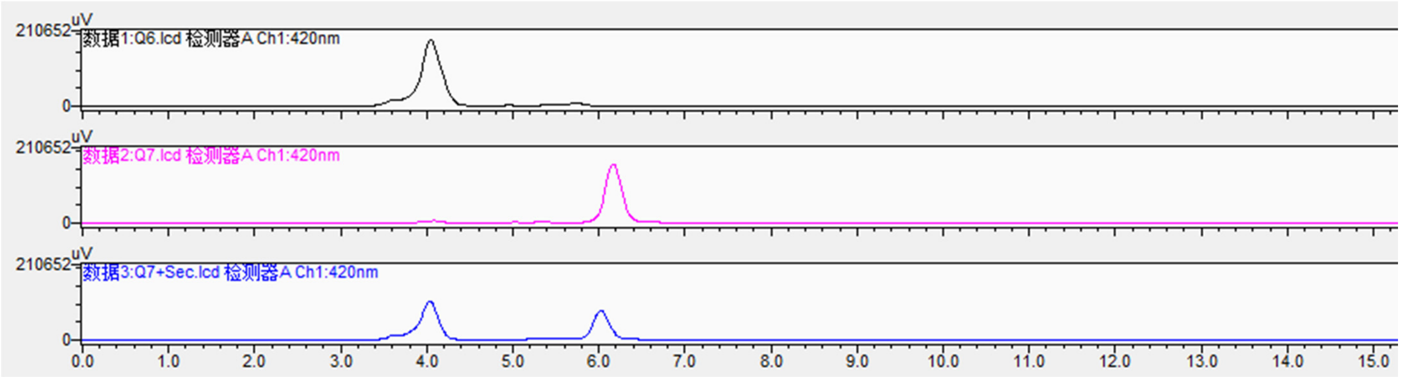

Figure S13 HPLC spectrum of compound **Q6** (black)**, Q7** (pink) and treating **Q7** with Sec (blue). Mobile phase: MeOH : H_2O = 7:3, UV detection wavelength: 420nm.

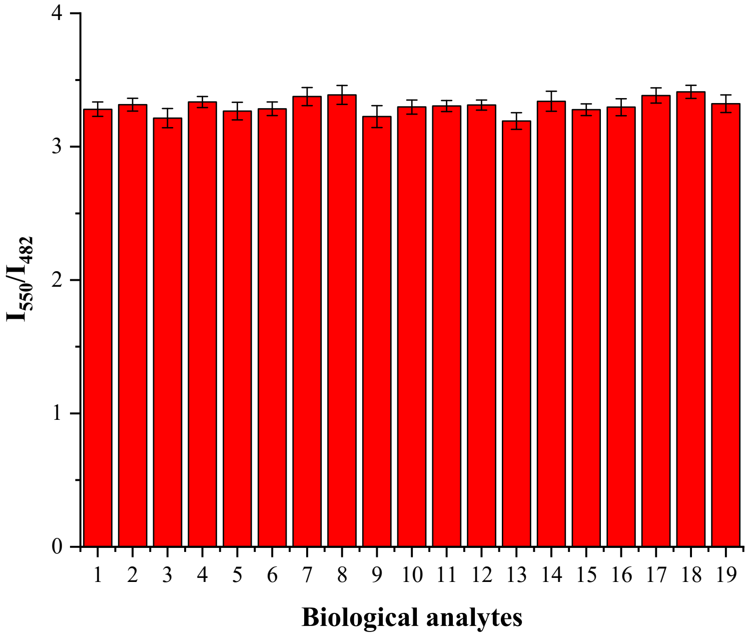

Figure S14 Fluorescent intensity ratios I_{550}/I_{482} responses of 1 μM **Q7** at 614 nm to Sec (40 μM) in the presence of various biological analytes (1 mM) in PBS (10 mM, pH 7.40, containing 1% DMSO as cosolvent). Legend: (1) Blank; (2) Hcy; (3) GSH; (4) Cys; (5) Pro; (6) Glu; (7) Asp; (8) Phe; (9) Thr; (10) Val, (11) Leu, (12) Arg; (13) Ile; (14) Ser; (15) Trp; (16) Lys; (17) His; (18) Na_2SeO_3; (19) Na_2Se. Excitation at 400 nm. Each data was obtained 4 min after mixing.

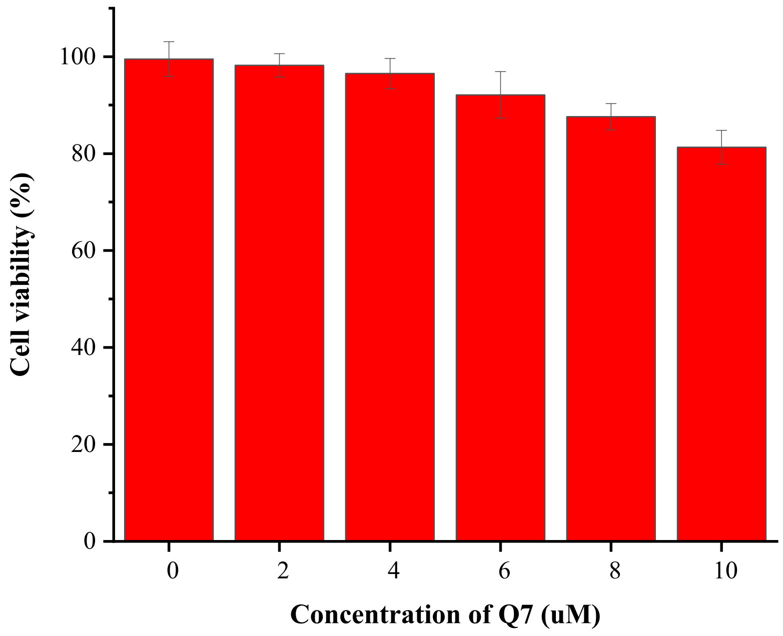

Figure S15 Cytotoxicity of A549 cells by standard MTT assay in the presence of sensor **Q7** (0~10 μM) at 37 °C.
